# Supplementary material for: Polymorphisms in Pfkelch13 domains before and after the introduction of artemisinin-based combination therapy in Southwest Nigeria
Source: PLoS One. 2025 Mar 31;20(3):e0316479. doi: 10.1371/journal.pone.0316479 (PMC11957316; doi:10.1371/journal.pone.0316479)
Supplement: Supporting information 2 — (ZIP) [file pone.0316479.s002.zip › 199KN1F_PREMIX_Plate_KELCH1_A07.pdf]

Samples: 13664
Bases: 839
Average spacing: 17

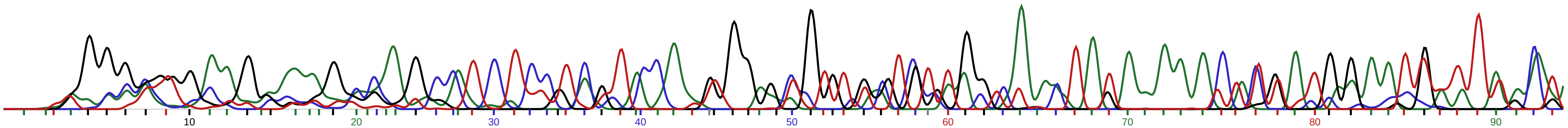

A A T A G G G G T G A A G A G A A A G A A C A A G C C A T C T C C G T C G T A C A A T - G G G A G C G T G T G G T C - T G G C A A C T A T A A A A A T T T T A T G G A A T G T T T A A C T

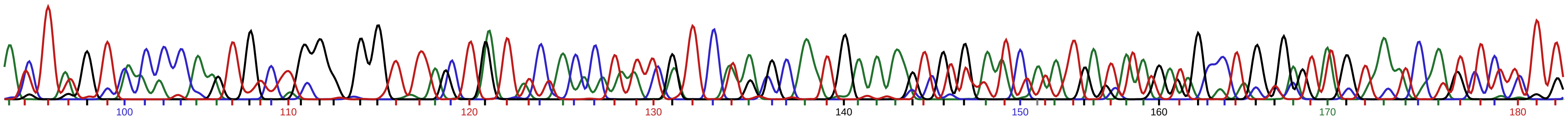

A T T T G T C C C C A A T G T C T G G G G G T T A C T G T T C A C C T T T G T C T A C C A T G A A A G T G G A T C - T A T G A T A A G T G C C T G G A T A G T A A C A T T C T T T

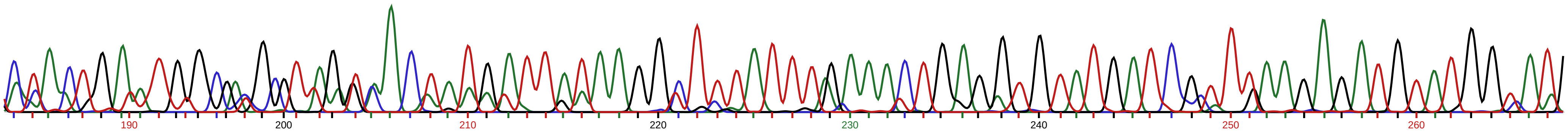

C T A C T G A T A T G T G C G T G G T T G T A A C T A T G A T T A T A A G G C T T T A T T T G A A A C T G A G G T G T A T G A T C G T T T A A G A G A T G T A T G G T A T

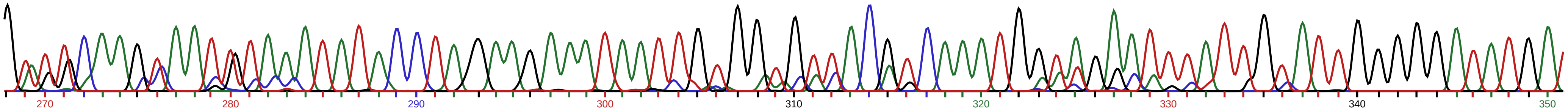

G T T T C A A G T A A T T T A A A T A T A C C T A G A A G A A A T A A T T G T G G T G T T A C G T C A A A T G G T A G A A T T T A T T G T A T T G G G G G A T A T G A

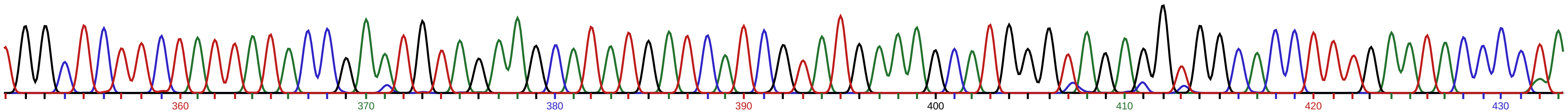

T G G C T C T T C T A T T A T A C C G A A T G T A G A A G C A T A T G A T C A T C G T A T G A A A G C A T G G G T A G A G G T G G C A C C T T T G A A T A C C C C T A

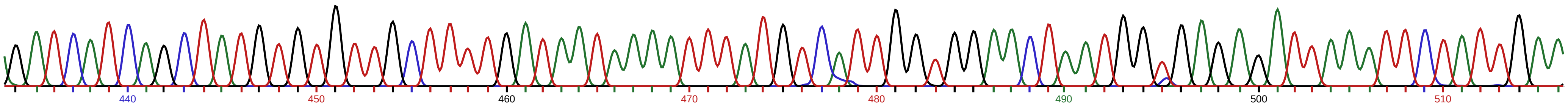

G A T C A T C A G C T A T G T G T G T T G C T T T T G A T A A T A A A A T T T A T G T C A T T G G T G G A A C T A A T G G T G A G A G A T T A A A T T C T A T T G A A

Samples: 13664
Bases: 839
Average spacing: 17

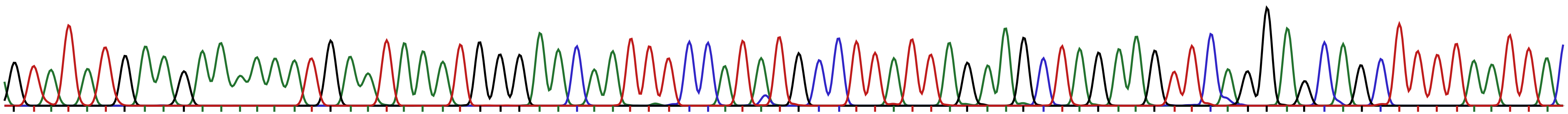

520 530 540 550 560 570 580 590

G T A T A T G A A G A A A A A A T G A A T A A A T G G G A A C A A T T T C C A T A T G C C T T A T T A G A A G C T A G A A G T T C A G G A G C A G C T T T T A A T T A

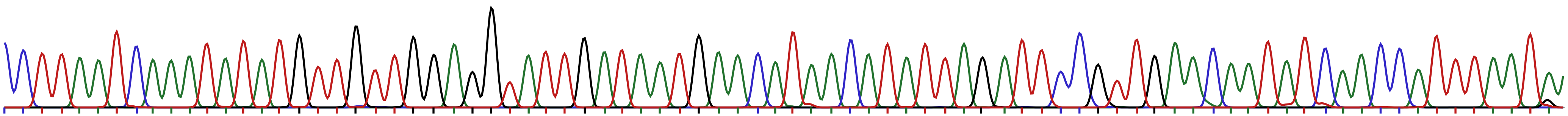

600 610 620 630 640 650 660 670 680

C C T T A A T C A A A T A T A T G T T G T T G G A G G T A T T G A T A A T G A A C A T A A C A T A T T A G A T T C C G T T G A A C A A T A T C A A C C A T T T A A T A

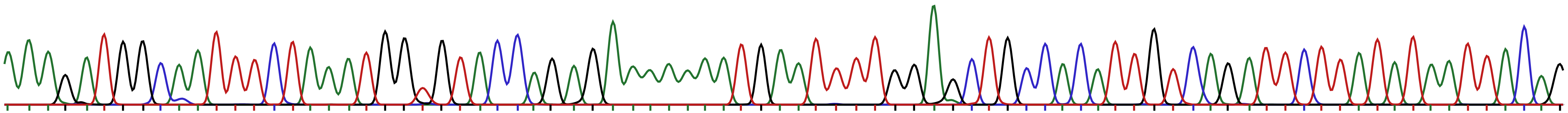

690 700 710 720 730 740 750 760

A A A G A T G G C A A T T T C T A A A T G G T G T A C C A G A G A A A A A A T G A A T T T T G G A G C T G C C A C A T T G T C A G A T T C T T A T A T A A T T A C A G

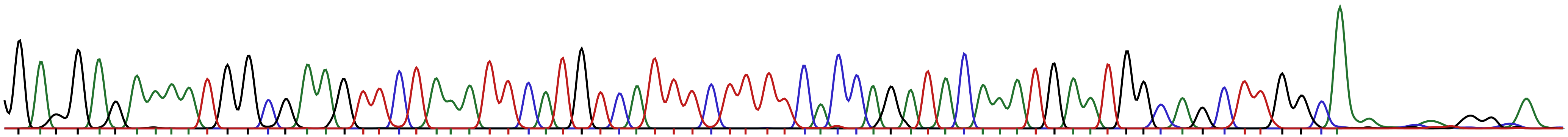

770 780 790 800 810 820 830

G A G G A G A A A A T G G C G A A G T T C T A A A T T C A T G T C A T T T C T T T T C A C C A G A T A C A A A T G A A T G G C A G C T T G G C A

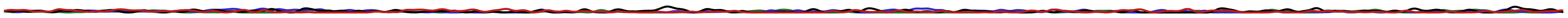

Samples:	13664
Bases:	839
Average spacing:	17
